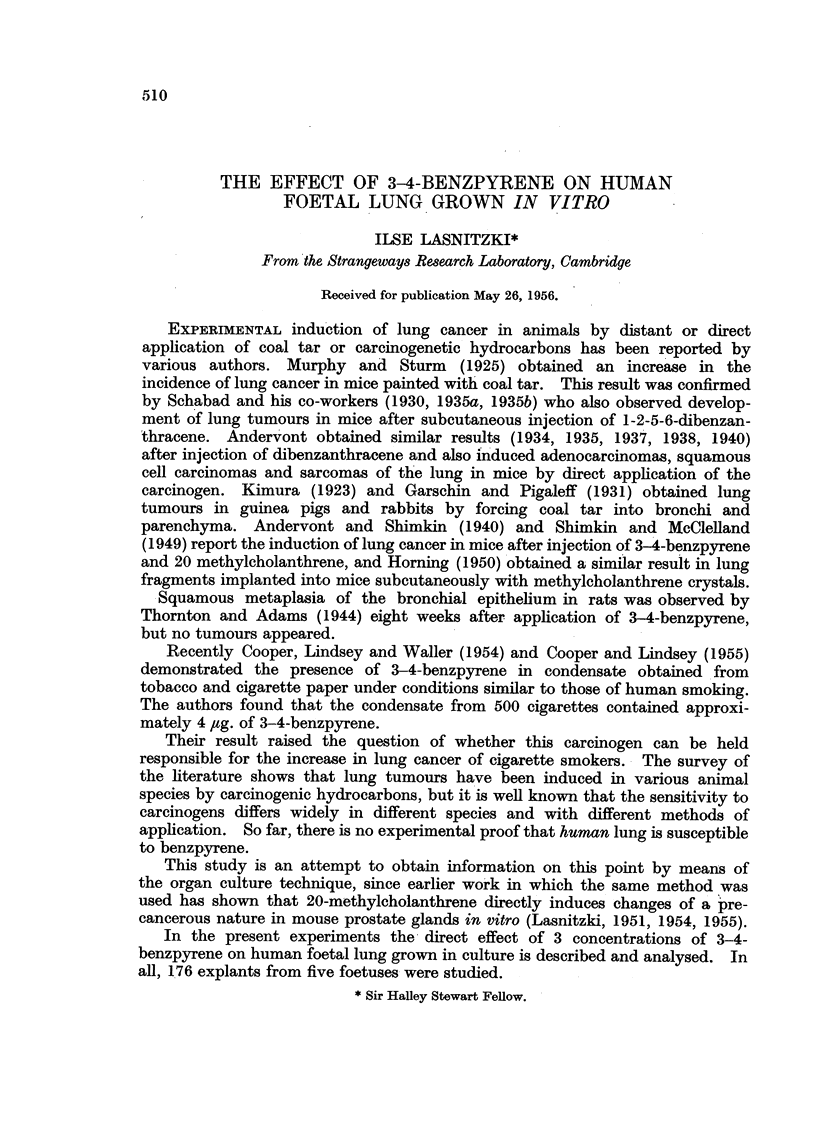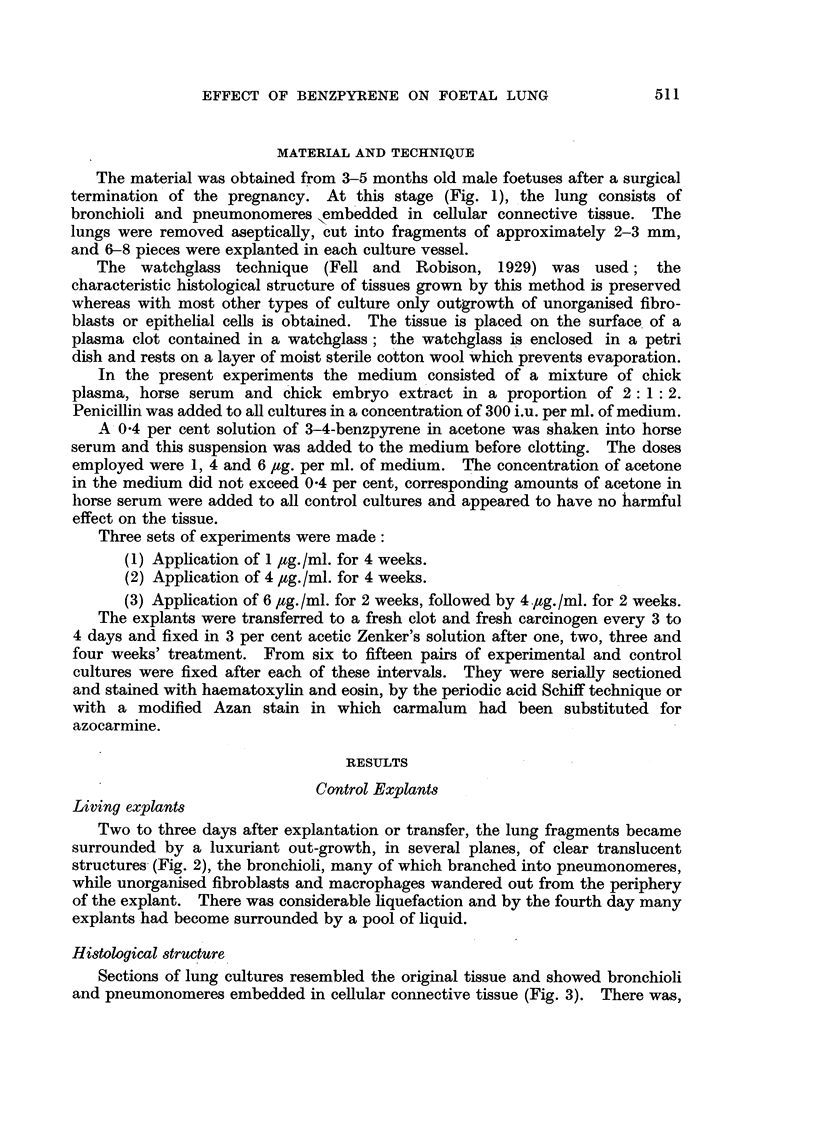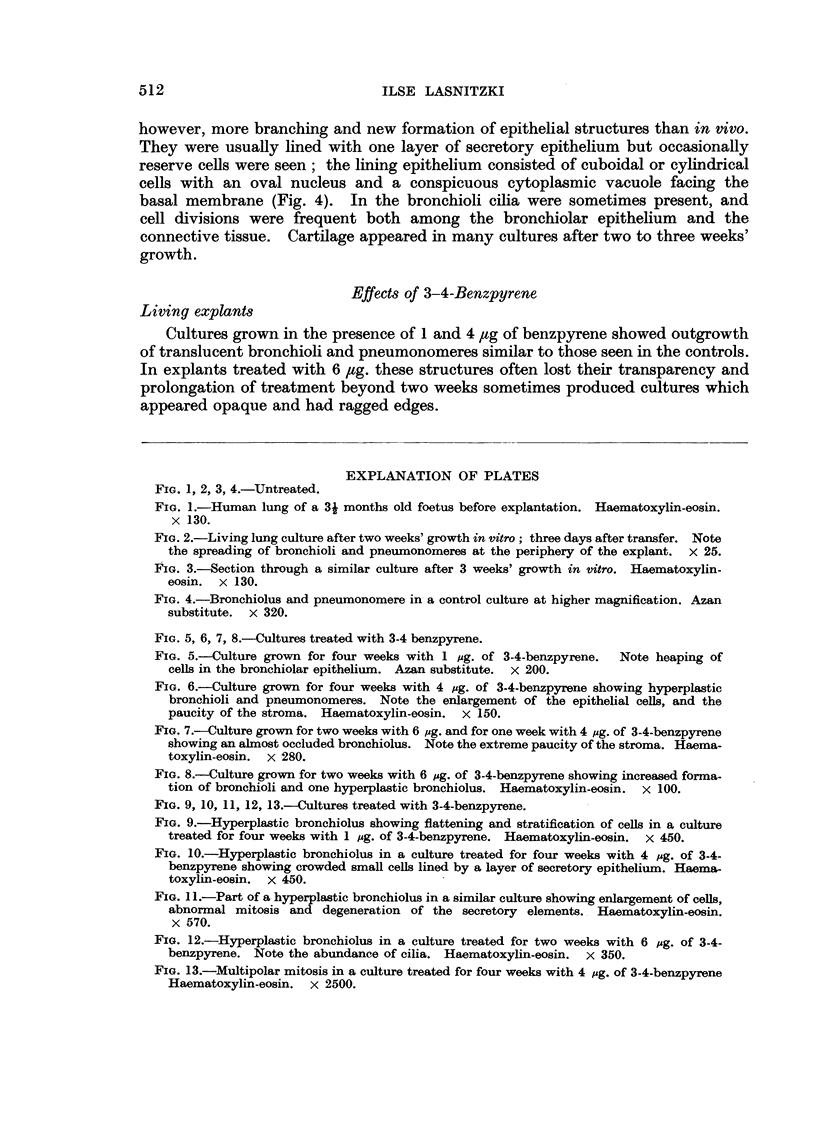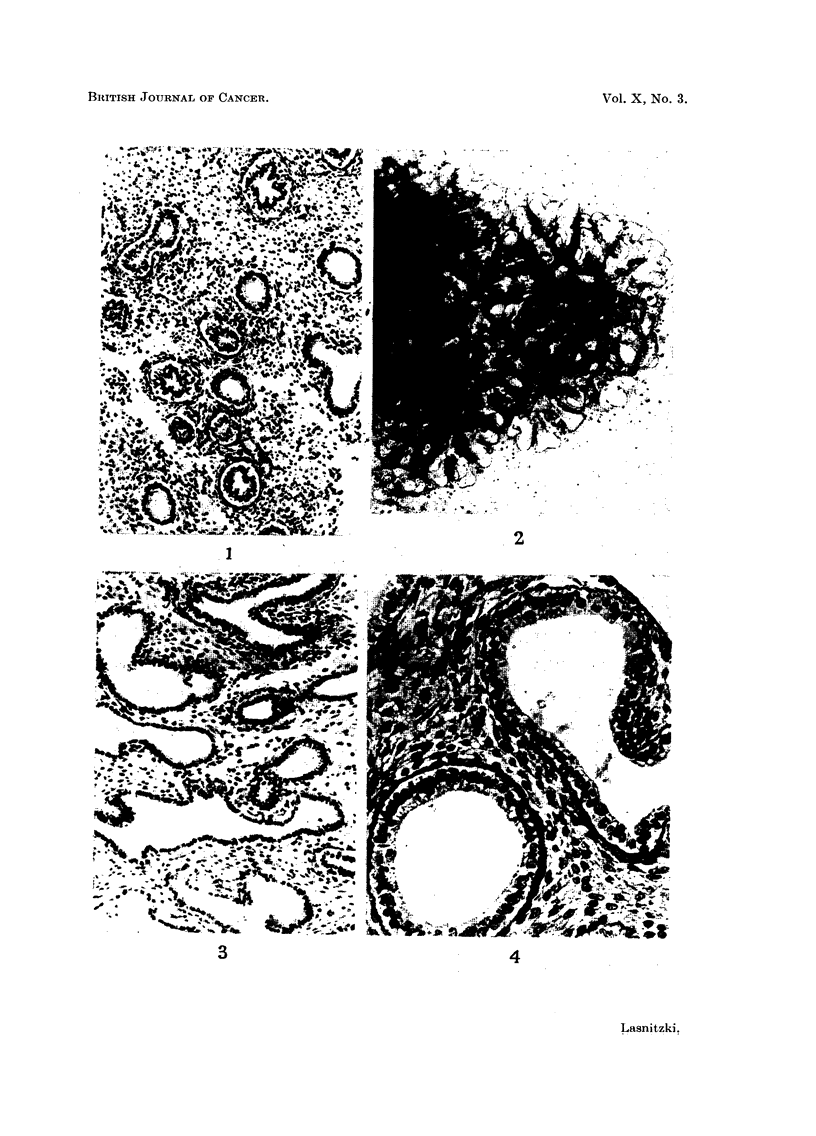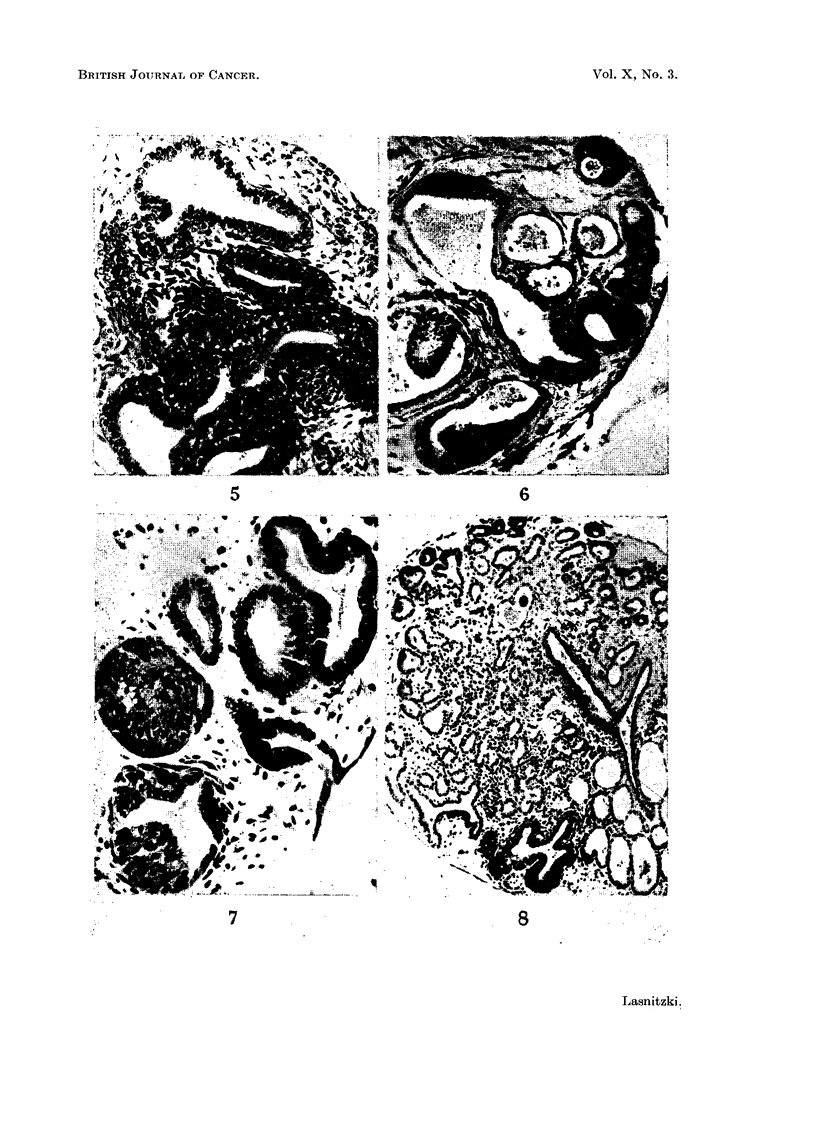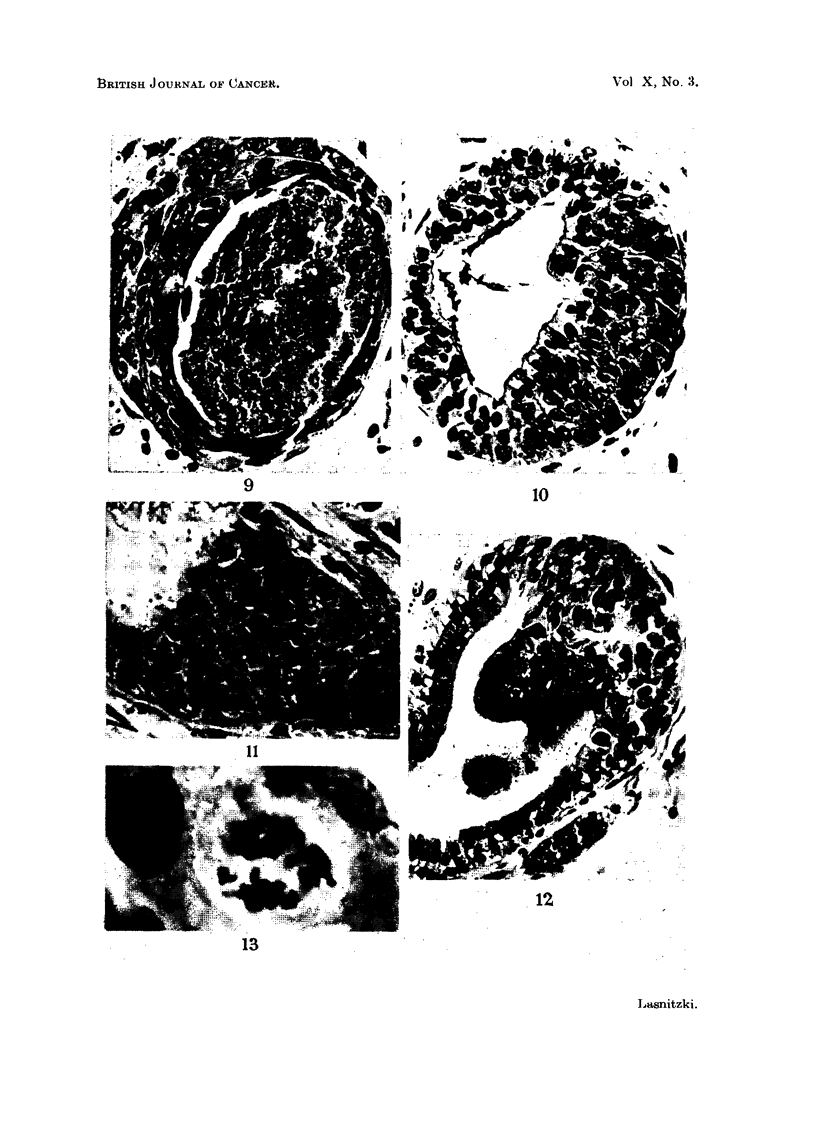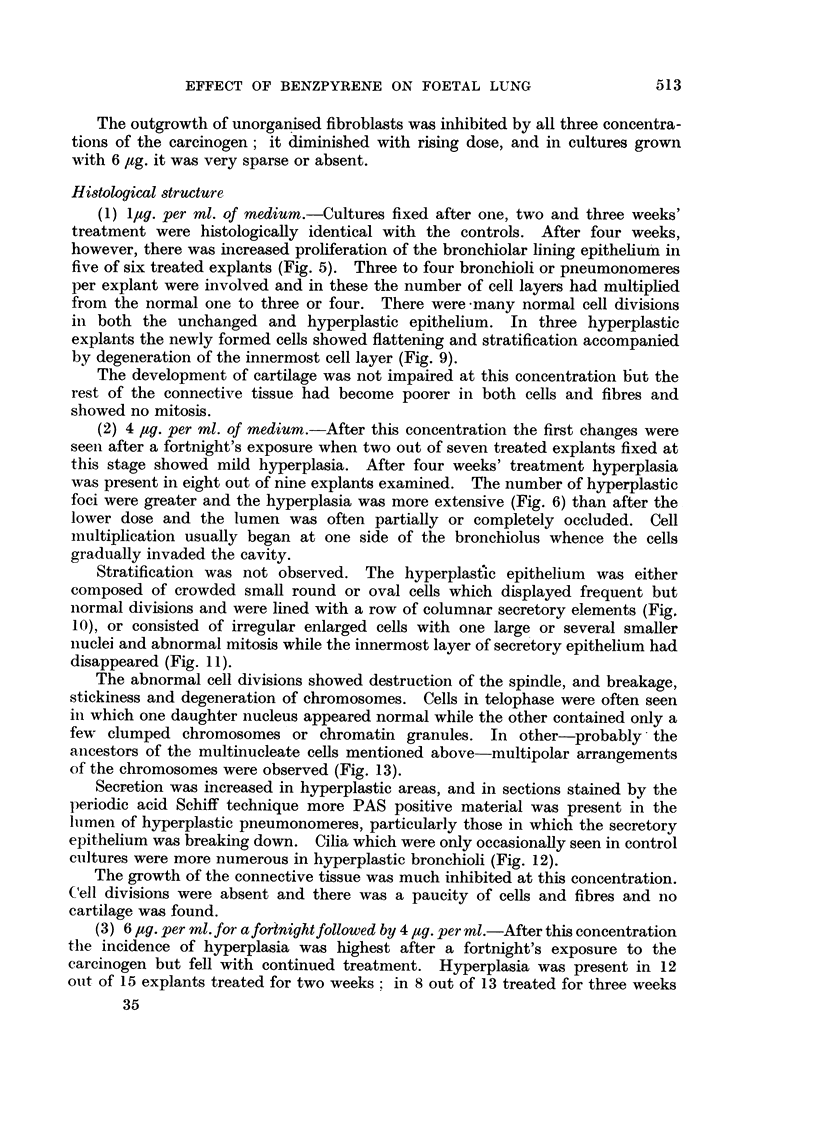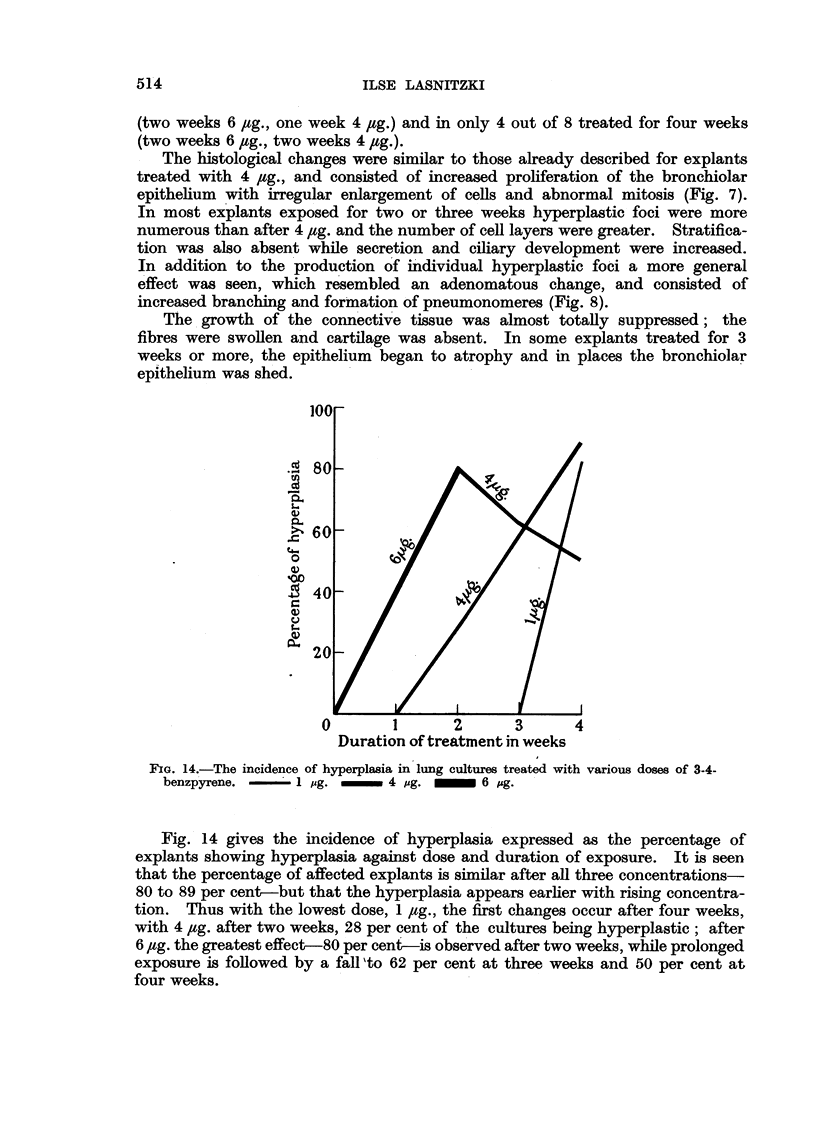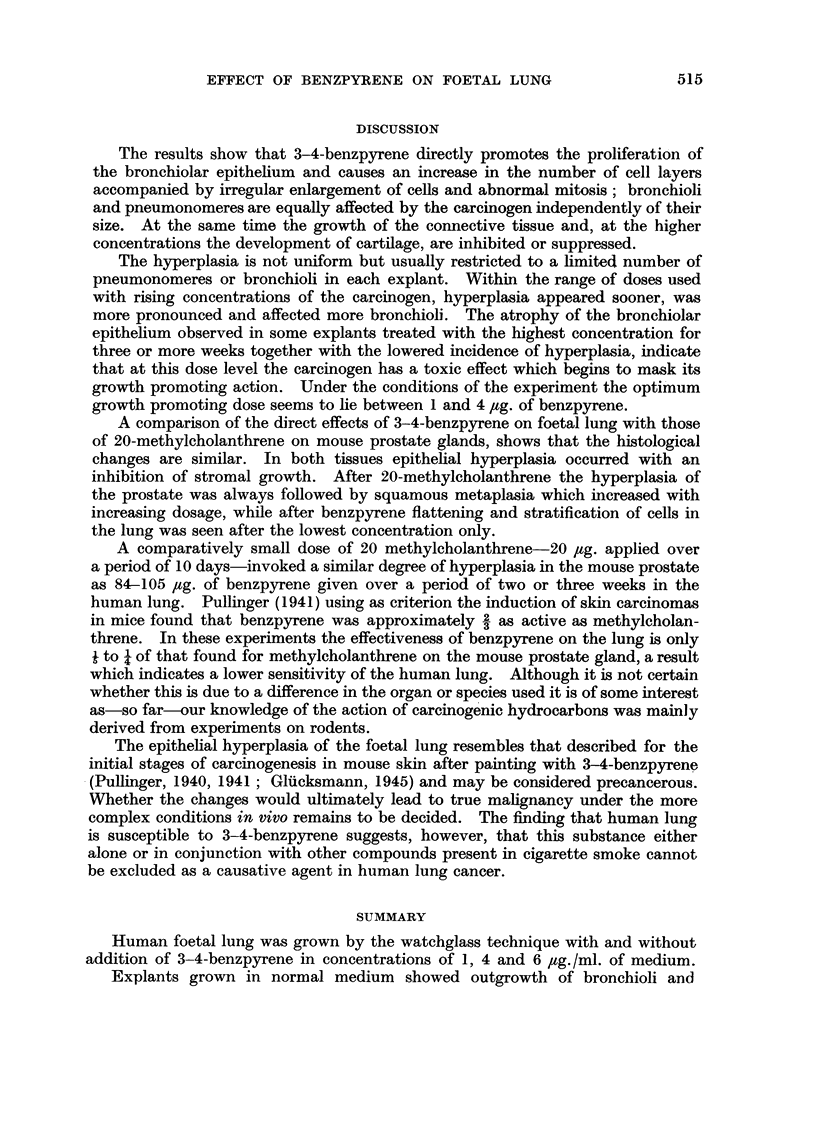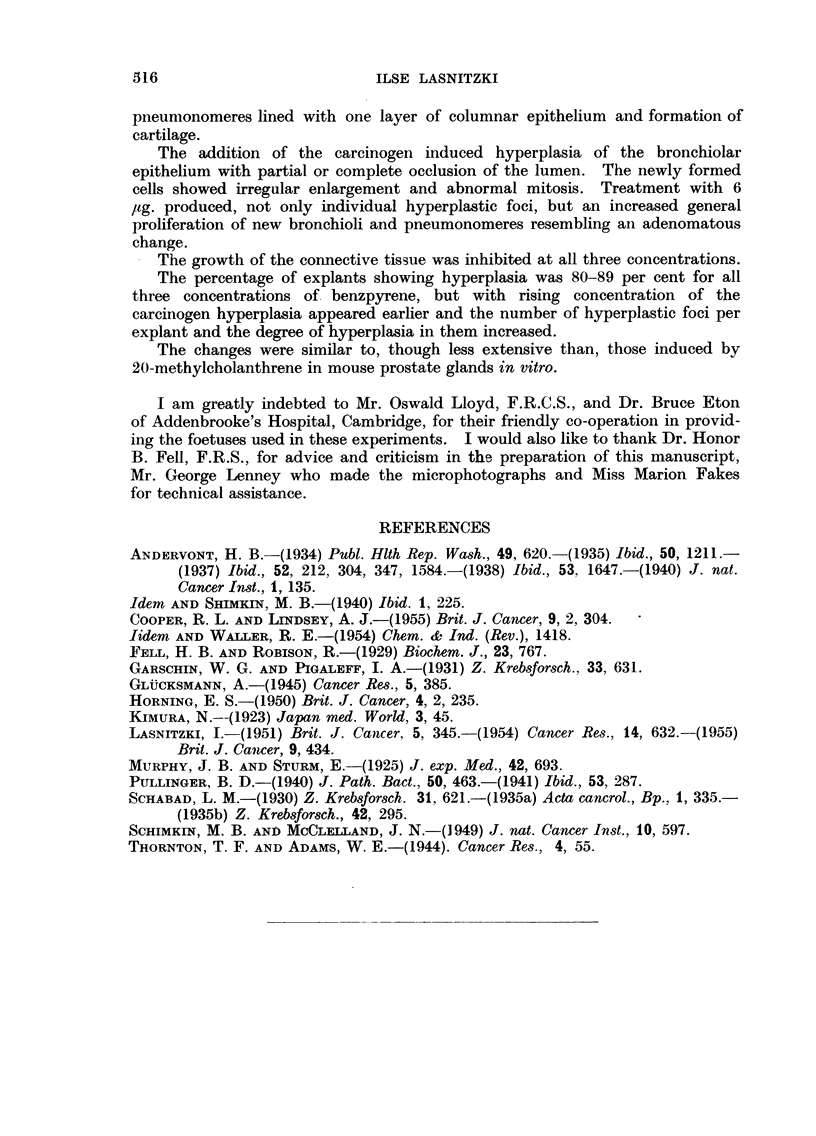# The Effect of 3-4-Benzpyrene on Human Foetal Lung Grown in vitro

**DOI:** 10.1038/bjc.1956.59

**Published:** 1956-09

**Authors:** Ilse Lasnitzki

## Abstract

**Images:**


### THE EFFECT OF 3–4-BENZPYRENE ON HUMAN FOETAL LUNG GROWN *IN VITRO*

ILSE LASNITZKI*

*From the Strangeways Research Laboratory, Cambridge*

Received for publication May 26, 1956.

EXPERIMENTAL induction of lung cancer in animals by distant or direct application of coal tar or carcinogenetic hydrocarbons has been reported by various authors. Murphy and Sturm (1925) obtained an increase in the incidence of lung cancer in mice painted with coal tar. This result was confirmed by Schabad and his co-workers (1930, 1935*a*, 1935*b*) who also observed development of lung tumours in mice after subcutaneous injection of 1-2-5-6-dibenzanthracene. Andervont obtained similar results (1934, 1935, 1937, 1938, 1940) after injection of dibenzanthracene and also induced adenocarcinomas, squamous cell carcinomas and sarcomas of the lung in mice by direct application of the carcinogen. Kimura (1923) and Garschin and Pigaleff (1931) obtained lung tumours in guinea pigs and rabbits by forcing coal tar into bronchi and parenchyma. Andervont and Shimkin (1940) and Shimkin and McClelland (1949) report the induction of lung cancer in mice after injection of 3–4-benzpyrene and 20 methylcholanthrene, and Horning (1950) obtained a similar result in lung fragments implanted into mice subcutaneously with methylcholanthrene crystals.

Squamous metaplasia of the bronchial epithelium in rats was observed by Thornton and Adams (1944) eight weeks after application of 3–4-benzpyrene, but no tumours appeared.

Recently Cooper, Lindsey and Waller (1954) and Cooper and Lindsey (1955) demonstrated the presence of 3–4-benzpyrene in condensate obtained from tobacco and cigarette paper under conditions similar to those of human smoking. The authors found that the condensate from 500 cigarettes contained approximately 4 $\mu$g. of 3–4-benzpyrene.

Their result raised the question of whether this carcinogen can be held responsible for the increase in lung cancer of cigarette smokers. The survey of the literature shows that lung tumours have been induced in various animal species by carcinogenic hydrocarbons, but it is well known that the sensitivity to carcinogens differs widely in different species and with different methods of application. So far, there is no experimental proof that *human* lung is susceptible to benzpyrene.

This study is an attempt to obtain information on this point by means of the organ culture technique, since earlier work in which the same method was used has shown that 20-methylcholanthrene directly induces changes of a precancerous nature in mouse prostate glands *in vitro* (Lasnitzki, 1951, 1954, 1955).

In the present experiments the direct effect of 3 concentrations of 3–4-benzpyrene on human foetal lung grown in culture is described and analysed. In all, 176 explants from five foetuses were studied.

* Sir Halley Stewart Fellow.

### MATERIAL AND TECHNIQUE

The material was obtained from 3–5 months old male foetuses after a surgical termination of the pregnancy. At this stage (Fig. 1), the lung consists of bronchioli and pneumonomeres embedded in cellular connective tissue. The lungs were removed aseptically, cut into fragments of approximately 2–3 mm, and 6–8 pieces were explanted in each culture vessel.

The watchglass technique (Fell and Robison, 1929) was used; the characteristic histological structure of tissues grown by this method is preserved whereas with most other types of culture only outgrowth of unorganised fibroblasts or epithelial cells is obtained. The tissue is placed on the surface of a plasma clot contained in a watchglass; the watchglass is enclosed in a petri dish and rests on a layer of moist sterile cotton wool which prevents evaporation.

In the present experiments the medium consisted of a mixture of chick plasma, horse serum and chick embryo extract in a proportion of 2 : 1 : 2. Penicillin was added to all cultures in a concentration of 300 i.u. per ml. of medium.

A 0·4 per cent solution of 3–4-benzpyrene in acetone was shaken into horse serum and this suspension was added to the medium before clotting. The doses employed were 1, 4 and 6 $\mu$g. per ml. of medium. The concentration of acetone in the medium did not exceed 0·4 per cent, corresponding amounts of acetone in horse serum were added to all control cultures and appeared to have no harmful effect on the tissue.

Three sets of experiments were made:

(1) Application of 1 $\mu$g./ml. for 4 weeks.

(2) Application of 4 $\mu$g./ml. for 4 weeks.

(3) Application of 6 $\mu$g./ml. for 2 weeks, followed by 4 $\mu$g./ml. for 2 weeks.

The explants were transferred to a fresh clot and fresh carcinogen every 3 to 4 days and fixed in 3 per cent acetic Zenker's solution after one, two, three and four weeks' treatment. From six to fifteen pairs of experimental and control cultures were fixed after each of these intervals. They were serially sectioned and stained with haematoxylin and eosin, by the periodic acid Schiff technique or with a modified Azan stain in which carmalum had been substituted for azocarmine.

### RESULTS

### *Control Explants*

#### *Living explants*

Two to three days after explantation or transfer, the lung fragments became surrounded by a luxuriant out-growth, in several planes, of clear translucent structures (Fig. 2), the bronchioli, many of which branched into pneumonomeres, while unorganised fibroblasts and macrophages wandered out from the periphery of the explant. There was considerable liquefaction and by the fourth day many explants had become surrounded by a pool of liquid.

#### *Histological structure*

Sections of lung cultures resembled the original tissue and showed bronchioli and pneumonomeres embedded in cellular connective tissue (Fig. 3). There was, however, more branching and new formation of epithelial structures than *in vivo*. They were usually lined with one layer of secretory epithelium but occasionally reserve cells were seen ; the lining epithelium consisted of cuboidal or cylindrical cells with an oval nucleus and a conspicuous cytoplasmic vacuole facing the basal membrane (Fig. 4). In the bronchioli cilia were sometimes present, and cell divisions were frequent both among the bronchiolar epithelium and the connective tissue. Cartilage appeared in many cultures after two to three weeks' growth.

### *Effects of* 3–4-*Benzpyrene*

*Living explants*

Cultures grown in the presence of 1 and 4 $\mu$g of benzpyrene showed outgrowth of translucent bronchioli and pneumonomeres similar to those seen in the controls. In explants treated with 6 $\mu$g. these structures often lost their transparency and prolongation of treatment beyond two weeks sometimes produced cultures which appeared opaque and had ragged edges.

---

EXPLANATION OF PLATES

FIG. 1, 2, 3, 4.—Untreated.

FIG. 1.—Human lung of a 3½ months old foetus before explantation. Haematoxylin-eosin. × 130.

FIG. 2.—Living lung culture after two weeks' growth *in vitro* ; three days after transfer. Note the spreading of bronchioli and pneumonomeres at the periphery of the explant. × 25.

FIG. 3.—Section through a similar culture after 3 weeks' growth *in vitro*. Haematoxylin-eosin. × 130.

FIG. 4.—Bronchiolus and pneumonomere in a control culture at higher magnification. Azan substitute. × 320.

FIG. 5, 6, 7, 8.—Cultures treated with 3-4 benzpyrene.

FIG. 5.—Culture grown for four weeks with 1 $\mu$g. of 3-4-benzpyrene. Note heaping of cells in the bronchiolar epithelium. Azan substitute. × 200.

FIG. 6.—Culture grown for four weeks with 4 $\mu$g. of 3-4-benzpyrene showing hyperplastic bronchioli and pneumonomeres. Note the enlargement of the epithelial cells, and the paucity of the stroma. Haematoxylin-eosin. × 150.

FIG. 7.—Culture grown for two weeks with 6 $\mu$g. and for one week with 4 $\mu$g. of 3-4-benzpyrene showing an almost occluded bronchiolus. Note the extreme paucity of the stroma. Haematoxylin-eosin. × 280.

FIG. 8.—Culture grown for two weeks with 6 $\mu$g. of 3-4-benzpyrene showing increased formation of bronchioli and one hyperplastic bronchiolus. Haematoxylin-eosin. × 100.

FIG. 9, 10, 11, 12, 13.—Cultures treated with 3-4-benzpyrene.

FIG. 9.—Hyperplastic bronchiolus showing flattening and stratification of cells in a culture treated for four weeks with 1 $\mu$g. of 3-4-benzpyrene. Haematoxylin-eosin. × 450.

FIG. 10.—Hyperplastic bronchiolus in a culture treated for four weeks with 4 $\mu$g. of 3-4-benzpyrene showing crowded small cells lined by a layer of secretory epithelium. Haematoxylin-eosin. × 450.

FIG. 11.—Part of a hyperplastic bronchiolus in a similar culture showing enlargement of cells, abnormal mitosis and degeneration of the secretory elements. Haematoxylin-eosin. × 570.

FIG. 12.—Hyperplastic bronchiolus in a culture treated for two weeks with 6 $\mu$g. of 3-4-benzpyrene. Note the abundance of cilia. Haematoxylin-eosin. × 350.

FIG. 13.—Multipolar mitosis in a culture treated for four weeks with 4 $\mu$g. of 3-4-benzpyrene Haematoxylin-eosin. × 2500.

1

2

3

4

5

6

7

8

9

10

11

12

13

Lasnitzki.

The outgrowth of unorganised fibroblasts was inhibited by all three concentrations of the carcinogen ; it diminished with rising dose, and in cultures grown with 6 $\mu$g. it was very sparse or absent.

*Histological structure*

(1) 1$\mu$*g. per ml. of medium.*—Cultures fixed after one, two and three weeks' treatment were histologically identical with the controls. After four weeks, however, there was increased proliferation of the bronchiolar lining epithelium in five of six treated explants (Fig. 5). Three to four bronchioli or pneumonomeres per explant were involved and in these the number of cell layers had multiplied from the normal one to three or four. There were many normal cell divisions in both the unchanged and hyperplastic epithelium. In three hyperplastic explants the newly formed cells showed flattening and stratification accompanied by degeneration of the innermost cell layer (Fig. 9).

The development of cartilage was not impaired at this concentration but the rest of the connective tissue had become poorer in both cells and fibres and showed no mitosis.

(2) 4 $\mu$*g. per ml. of medium.*—After this concentration the first changes were seen after a fortnight's exposure when two out of seven treated explants fixed at this stage showed mild hyperplasia. After four weeks' treatment hyperplasia was present in eight out of nine explants examined. The number of hyperplastic foci were greater and the hyperplasia was more extensive (Fig. 6) than after the lower dose and the lumen was often partially or completely occluded. Cell multiplication usually began at one side of the bronchiolus whence the cells gradually invaded the cavity.

Stratification was not observed. The hyperplastic epithelium was either composed of crowded small round or oval cells which displayed frequent but normal divisions and were lined with a row of columnar secretory elements (Fig. 10), or consisted of irregular enlarged cells with one large or several smaller nuclei and abnormal mitosis while the innermost layer of secretory epithelium had disappeared (Fig. 11).

The abnormal cell divisions showed destruction of the spindle, and breakage, stickiness and degeneration of chromosomes. Cells in telophase were often seen in which one daughter nucleus appeared normal while the other contained only a few clumped chromosomes or chromatin granules. In other—probably the ancestors of the multinucleate cells mentioned above—multipolar arrangements of the chromosomes were observed (Fig. 13).

Secretion was increased in hyperplastic areas, and in sections stained by the periodic acid Schiff technique more PAS positive material was present in the lumen of hyperplastic pneumonomeres, particularly those in which the secretory epithelium was breaking down. Cilia which were only occasionally seen in control cultures were more numerous in hyperplastic bronchioli (Fig. 12).

The growth of the connective tissue was much inhibited at this concentration. Cell divisions were absent and there was a paucity of cells and fibres and no cartilage was found.

(3) 6 $\mu$*g. per ml. for a fortnight followed by* 4 $\mu$*g. per ml.*—After this concentration the incidence of hyperplasia was highest after a fortnight's exposure to the carcinogen but fell with continued treatment. Hyperplasia was present in 12 out of 15 explants treated for two weeks ; in 8 out of 13 treated for three weeks

(two weeks 6 μg., one week 4 μg.) and in only 4 out of 8 treated for four weeks (two weeks 6 μg., two weeks 4 μg.).

The histological changes were similar to those already described for explants treated with 4 μg., and consisted of increased proliferation of the bronchiolar epithelium with irregular enlargement of cells and abnormal mitosis (Fig. 7). In most explants exposed for two or three weeks hyperplastic foci were more numerous than after 4 μg. and the number of cell layers were greater. Stratification was also absent while secretion and ciliary development were increased. In addition to the production of individual hyperplastic foci a more general effect was seen, which resembled an adenomatous change, and consisted of increased branching and formation of pneumonomeres (Fig. 8).

The growth of the connective tissue was almost totally suppressed; the fibres were swollen and cartilage was absent. In some explants treated for 3 weeks or more, the epithelium began to atrophy and in places the bronchiolar epithelium was shed.

Fig. 14.—The incidence of hyperplasia in lung cultures treated with various doses of 3-4-benzpyrene. ——— 1 μg. ——— 4 μg. ——— 6 μg.

Fig. 14 gives the incidence of hyperplasia expressed as the percentage of explants showing hyperplasia against dose and duration of exposure. It is seen that the percentage of affected explants is similar after all three concentrations—80 to 89 per cent—but that the hyperplasia appears earlier with rising concentration. Thus with the lowest dose, 1 μg., the first changes occur after four weeks, with 4 μg. after two weeks, 28 per cent of the cultures being hyperplastic; after 6 μg. the greatest effect—80 per cent—is observed after two weeks, while prolonged exposure is followed by a fall to 62 per cent at three weeks and 50 per cent at four weeks.

### DISCUSSION

The results show that 3–4-benzpyrene directly promotes the proliferation of the bronchiolar epithelium and causes an increase in the number of cell layers accompanied by irregular enlargement of cells and abnormal mitosis ; bronchioli and pneumonomeres are equally affected by the carcinogen independently of their size. At the same time the growth of the connective tissue and, at the higher concentrations the development of cartilage, are inhibited or suppressed.

The hyperplasia is not uniform but usually restricted to a limited number of pneumonomeres or bronchioli in each explant. Within the range of doses used with rising concentrations of the carcinogen, hyperplasia appeared sooner, was more pronounced and affected more bronchioli. The atrophy of the bronchiolar epithelium observed in some explants treated with the highest concentration for three or more weeks together with the lowered incidence of hyperplasia, indicate that at this dose level the carcinogen has a toxic effect which begins to mask its growth promoting action. Under the conditions of the experiment the optimum growth promoting dose seems to lie between 1 and 4 $\mu$g. of benzpyrene.

A comparison of the direct effects of 3–4-benzpyrene on foetal lung with those of 20-methylcholanthrene on mouse prostate glands, shows that the histological changes are similar. In both tissues epithelial hyperplasia occurred with an inhibition of stromal growth. After 20-methylcholanthrene the hyperplasia of the prostate was always followed by squamous metaplasia which increased with increasing dosage, while after benzpyrene flattening and stratification of cells in the lung was seen after the lowest concentration only.

A comparatively small dose of 20 methylcholanthrene—20 $\mu$g. applied over a period of 10 days—invoked a similar degree of hyperplasia in the mouse prostate as 84–105 $\mu$g. of benzpyrene given over a period of two or three weeks in the human lung. Pullinger (1941) using as criterion the induction of skin carcinomas in mice found that benzpyrene was approximately $\frac{2}{3}$ as active as methylcholanthrene. In these experiments the effectiveness of benzpyrene on the lung is only $\frac{1}{5}$ to $\frac{1}{4}$ of that found for methylcholanthrene on the mouse prostate gland, a result which indicates a lower sensitivity of the human lung. Although it is not certain whether this is due to a difference in the organ or species used it is of some interest as—so far—our knowledge of the action of carcinogenic hydrocarbons was mainly derived from experiments on rodents.

The epithelial hyperplasia of the foetal lung resembles that described for the initial stages of carcinogenesis in mouse skin after painting with 3–4-benzpyrene (Pullinger, 1940, 1941 ; Glücksmann, 1945) and may be considered precancerous. Whether the changes would ultimately lead to true malignancy under the more complex conditions *in vivo* remains to be decided. The finding that human lung is susceptible to 3–4-benzpyrene suggests, however, that this substance either alone or in conjunction with other compounds present in cigarette smoke cannot be excluded as a causative agent in human lung cancer.

### SUMMARY

Human foetal lung was grown by the watchglass technique with and without addition of 3–4-benzpyrene in concentrations of 1, 4 and 6 $\mu$g./ml. of medium.

Explants grown in normal medium showed outgrowth of bronchioli and pneumonomeres lined with one layer of columnar epithelium and formation of cartilage.

The addition of the carcinogen induced hyperplasia of the bronchiolar epithelium with partial or complete occlusion of the lumen. The newly formed cells showed irregular enlargement and abnormal mitosis. Treatment with 6 $\mu$g. produced, not only individual hyperplastic foci, but an increased general proliferation of new bronchioli and pneumonomeres resembling an adenomatous change.

The growth of the connective tissue was inhibited at all three concentrations.

The percentage of explants showing hyperplasia was 80–89 per cent for all three concentrations of benzpyrene, but with rising concentration of the carcinogen hyperplasia appeared earlier and the number of hyperplastic foci per explant and the degree of hyperplasia in them increased.

The changes were similar to, though less extensive than, those induced by 20-methylcholanthrene in mouse prostate glands *in vitro.*

I am greatly indebted to Mr. Oswald Lloyd, F.R.C.S., and Dr. Bruce Eton of Addenbrooke's Hospital, Cambridge, for their friendly co-operation in providing the foetuses used in these experiments. I would also like to thank Dr. Honor B. Fell, F.R.S., for advice and criticism in the preparation of this manuscript, Mr. George Lenney who made the microphotographs and Miss Marion Fakes for technical assistance.

### REFERENCES

ANDERVONT, H. B.—(1934) *Publ. Hlth Rep. Wash.*, **49**, 620.—(1935) *Ibid.*, **50**, 1211.—(1937) *Ibid.*, **52**, 212, 304, 347, 1584.—(1938) *Ibid.*, **53**, 1647.—(1940) *J. nat. Cancer Inst.*, **1**, 135.

*Idem* AND SHIMKIN, M. B.—(1940) *Ibid.* **1**, 225.

COOPER, R. L. AND LINDSEY, A. J.—(1955) *Brit. J. Cancer*, **9**, 2, 304.

*Iidem* AND WALLER, R. E.—(1954) *Chem. & Ind.* (*Rev.*), 1418.

FELL, H. B. AND ROBISON, R.—(1929) *Biochem. J.*, **23**, 767.

GARSCHIN, W. G. AND PIGALEFF, I. A.—(1931) *Z. Krebsforsch.*, **33**, 631.

GLÜCKSMANN, A.—(1945) *Cancer Res.*, **5**, 385.

HORNING, E. S.—(1950) *Brit. J. Cancer*, **4**, 2, 235.

KIMURA, N.—(1923) *Japan med. World*, **3**, 45.

LASNITZKI, I.—(1951) *Brit. J. Cancer*, **5**, 345.—(1954) *Cancer Res.*, **14**, 632.—(1955) *Brit. J. Cancer*, **9**, 434.

MURPHY, J. B. AND STURM, E.—(1925) *J. exp. Med.*, **42**, 693.

PULLINGER, B. D.—(1940) *J. Path. Bact.*, **50**, 463.—(1941) *Ibid.*, **53**, 287.

SCHABAD, L. M.—(1930) *Z. Krebsforsch.* **31**, 621.—(1935a) *Acta cancrol., Bp.*, **1**, 335.—(1935b) *Z. Krebsforsch.*, **42**, 295.

SCHIMKIN, M. B. AND MCCLELLAND, J. N.—(1949) *J. nat. Cancer Inst.*, **10**, 597.

THORNTON, T. F. AND ADAMS, W. E.—(1944). *Cancer Res.*, **4**, 55.